# A low-cost Behavioural Nudge and choice architecture intervention targeting school lunches increases children’s consumption of fruit: a cluster randomised trial

**DOI:** 10.1186/s12966-019-0773-x

**Published:** 2019-02-13

**Authors:** Mariel Marcano-Olivier, Ruth Pearson, Allycea Ruparell, Pauline J. Horne, Simon Viktor, Mihela Erjavec

**Affiliations:** The Centre for Activity and Eating Research, Bangor University, School of Psychology, Brigantia, Penrallt Road, Bangor (Gwynedd), Wales LL57 2AS UK

**Keywords:** Choice architecture, Behavioural nudges, Fruit, Plant-based foods, Consumption, School lunch, Cafeteria, Healthy eating, Children

## Abstract

**Background:**

Research has consistently indicated that most children do not consume sufficient fruit and vegetables to provide them with a healthy, balanced diet. This study set out to trial a simple, low-cost behavioural nudge intervention to encourage children to select and consume more fruit and vegetables with their lunchtime meal in a primary school cafeteria.

**Methods:**

Four primary schools were randomly allocated to either the control or the intervention condition and baseline data were collected over two days in each school. Following this, changes were made to the choice architecture of the school cafeterias in the intervention schools and maintained over a three-week period. The intervention included improved positioning and serving of fruit, accompanied by attractive labelling of both fruit and vegetables on offer. Next, data were collected over two days in each school, with menus matched in each instance between baseline and follow-up. We employed a validated and sensitive photographic method to estimate individual children’s (*N* = 176) consumption of vegetables, fruit, vitamin C, fibre, total sugars, and their overall calorie intake.

**Results:**

Significant increases were recorded in the intervention schools for children’s consumption of fruit, vitamin C, and fibre. No significant changes were observed in the control condition. The increases in fruit consumption were recorded in a large proportion of individual children, irrespective of their baseline consumption levels. No changes in vegetable consumption were observed in either condition.

**Conclusions:**

These results are the first to show that modest improvements to the choice architecture of school catering, and inclusion of behavioural nudges, can significantly increase fruit consumption, rather than just selection, in primary-age children. This has implications for the development of national and international strategies to promote healthy eating in schools.

**Trial registration:**

AsPredicted: 3943 05/02/2017. URL: https://aspredicted.org/see_one.php?a_id=3943

## Background

It is well established that a balanced diet high in fruit and vegetables supports positive developmental and health outcomes in children and adults, and ought to be promoted [1]. However, investigations into typical childhood eating patterns have consistently identified deficiencies in fruit and vegetable intake compared to international and local recommendations [2–4]. The UK Department of Health [5] has continued to advocate national-level efforts to encourage healthier childhood eating patterns, pledging to support healthy food provision in schools. While this is a positive step, provision does not equate to consumption, and structured behavioural programmes may be necessary to increase fruit and vegetable uptake.

Multicomponent interventions targeting school lunch nutrition have shown success [6, 7], but they are time costly and require substantial resources to implement effectively. This limits their potential for national-level support. Despite disappointingly few positive behaviour effects and poor intervention engagement [8], information-based interventions and social marketing campaigns, such as the ‘Change for Life’ [5], continue to receive governmental support and funding. It is perhaps not surprising that, at present, despite being familiar with the health benefits associated with a plant-rich diet [9], most primary-age children in the UK do not eat their recommended five portions of fruit and vegetables each day [10].

There is an emerging literature in the United States showing how low-cost interventions can improve dietary choices of adults and teenagers in a variety of canteen settings [11, 12]. These interventions manifest the typical properties of choice architecture interventions [13], altering the micro-environment of dining rooms to encourage healthy food selection outside of awareness, by increasing the salience of target foods and their convenience [14]. Such modifications to the environment are referred to as behavioural nudges. A smaller number of studies have examined the effectiveness of nudge interventions with younger children, in elementary school cafeterias [15–17]. Whilst many of these interventions reported success in increasing selection of target healthy food items, consumption was seldom measured, resulting in poor internal validity. For those studies that did measure consumption, procedural issues limited the conclusions that could be drawn from the data [18, 19]. The methodological shortcomings of these studies, detailed in an unpublished systematic review ( [20], currently under review), include the absence of a control group; lack of independently validated measures; single-day data collection; and use of group data.

The present study addressed these shortcomings and extended the existing literature to the UK school settings, where the mid-day meal is consumed by all children in a dining room. A small team of four caterers prepare a hot meal for children (with alternative options available for those with special dietary requirements), serve this with a selection of vegetables to those children whose carers pay for school meals, and to children with low-income families who receive meals for free (approximately half of school pupils bring their own home-provided lunch, and were not included in the present study).

To our knowledge, this was the first controlled experimental evaluation of the changes in individual children’s consumption that may be engendered by a low-cost behavioural nudge intervention in primary-school dining environments. A cluster randomised design was utilised, as the intervention was delivered at the school-level, rather than the individual level; schools were randomly allocated to experimental conditions, and striated sampling ensured that participants’ ages spanned the primary-school range. Individual children’s consumption was measured using an independently validated digital photography method [21–23], which yielded fine-grained estimates of the weight of fruit and vegetables that the children consumed, and the nutrient content of their lunches as a whole, enabling us to identify intervention success at both cohort and individual level. Pre- and post-measures were taken over two days in each instance, to accommodate variability of children’s daily choices and menu differences.

## Method

### Aim

This study was designed to investigate the effectiveness of a behavioural nudge intervention encouraging children to consume more fruit and vegetables with their lunchtime meal in the school cafeteria.

### Trial design

A cluster randomised design was utilised. Primary schools in North Wales catered for by a single school catering company comprised each of the four clusters, of which two were randomly allocated to the intervention condition, and two to the control condition. Schools were randomly allocated to conditions by the lead author using a coin-flip method prior to any data collection.

### Participants

Following institutional ethical approval and the distribution of opt-out consent forms one week prior to randomisation and study commencement, 176 children took part (intervention *n* = 86, control *n* = 90). No parent opted-out, and every child contributed data to the final sample. Both conditions were gender balanced (40 females in the intervention condition and 49 in the control condition) and represented the full primary school age span (with 33 children in year 1; 45 in year 2; 22 in year 3; 21 in year 4; 46 in year 5; and 9 in year 6). Participants were of a predominantly Caucasian origin, reflecting the demographics of the region.

### Materials

Four digital cameras (Fujifilm Finepix, 16 M pixels, Model no. AX650), positioned on tripod stands (Tiffen Davis and Sanford, Vista EXPLORERV 60-Inch Tripod), with tape measures and protractors to ensure correct setup, were used to collect consumption data. Food items were displayed on plastic school dinner trays. White self-adhesive participant identification labels were attached to red metallic wrist bands given to each participant to wear during lunchtime, and to the tray for later coding of the food and waste in each photograph.

### Intervention procedure

#### Behavioural nudges

Several changes were made in the cafeterias of the intervention schools; no changes were made in the control schools. The choice architecture of the cafeteria was changed to include five behavioral nudges that have been used in previous research.

1. *Advertisements.* Brightly coloured posters advertising, “A Spring of Fruit and Vegetables”, displaying fruit and vegetables and cartoon characters of children enjoying these foods, were placed around the dinner hall, encouraging children to, “Let fruit and vegetables put a spring in your step”. At the beginning of the dinner queue, a further wipe-clean poster advertised the “vegetable of the day”, using new attractive names. All materials were presented bilingually in English and Welsh.
2. *Attractive names.* For each fruit or vegetable available to buy over the data-collection and intervention period, a new and exciting name was created. Examples included “Dinosaur Tree Broccoli”, “Anti Sneeze Peas”, and “Superpower Satsumas”.
3. *Food labels.* Every fruit and vegetable was labeled. These wipe-clean labels were placed in or on the associated serving bowl, drawing attention to the food with its attractive name, an exciting picture, and a cartoon character.
4. *Attractive servings.* Whole fruit servings (available daily instead of, or in addition to, desserts in both schools) were replaced by sliced fruit, placed into colourful plastic bowls, and displayed on a cake stand at the end of the dinner queue.
5. *Fruit and veg first.* Where possible, the order of service was meant to be changed so that vegetables were served before the entrée or starchy side, and fruit was offered before the provided dessert. Catering staff were asked to encourage children to take a serving of vegetable and fruit with their meal.

##### Rationale for Nudge Selection

The five nudges employed were identified through systematic review [^20^, under review], typologies of which altered both properties and placement of the food items [13], to promote healthful consumption behavior outside of awareness. It was anticipated, due to its success in previous interventions, that slicing fruit into bite-sized pieces [23, 24] and serving them in brightly coloured “take away” bowls, would be the nudge that would be most likely to elicit significant behavior change, and that re-ordering service would prompt children to select a vegetable and fruit option [25], though this intervention does not consistently elicit significant change [26]. The remaining three nudges would aid in drawing children’s attention to this new presentation style. These more simple “advertisement” nudges were cheap to implement, and may not have been highly effective if implemented alone [27], but utilized together may have a cumulative influence on the children’s attention towards fruit and vegetables, resulting in the “presentation” nudges being more impactful.

#### Implementation

Following the initial visits to schools, it became apparent that the caterers already provided verbal encouragement for children’s selection of vegetables, and served salad daily, but did not do the same with fruit. Indeed, on most days, fruit was given only to those children who explicitly requested it, instead of their daily dessert, and hidden from children’s view, as it was selected so seldom that displaying it was considered an unnecessary use of canteen space. Therefore, the present intervention mostly targeted children’s fruit choices and consumption. Because of space limitations, the fruit stand had to be placed at the end of the dinner queue.

Prior to implementation, the intervention was discussed with caterers. Throughout the intervention, a researcher was present in the cafeteria at lunchtime to ensure intervention fidelity, and to minimise any disturbance for the catering staff. We established that typical daily provision of fruit consisted of bananas, satsumas, apples, or pears. These foods were therefore used in the intervention; we brought some extra when children’s consumption grew significantly, to avoid running out.

The intervention was implemented in two schools over a period of three weeks. It commenced in the week following the initial baseline data collection, and stopped after the follow-up data were collected. Throughout, we noted how many students took fruit from the stand. Daily selection of fruit pots ranged from 47 to 84 (out of a maximum of 132 students eating school dinners on any one day) over the course of the intervention. In some cases students helped themselves to fruit in addition to dessert; in others, the fruit replaced the dessert. The pots were available to all children who took school dinners, including those not participating in data collection.

#### Intervention cost

##### Resource costs

Tangible intervention resources provided to the schools included; colourful posters and food labels, which were printed and laminated by intervention staff (approximately £10 per school); colourful fruit pots and display stands (approximately £30 per school); and additional fruit (approximately £10 per school, per week).

##### Time commitment

Considering the simplicity of the intervention, very little time is required to train catering staff in the intervention protocol. On the first day of intervention implementation, approximately 30 min were taken to explain the concept of nudges, and their implementation. One member of the research team assisted and shadowed the catering staff for the duration of the intervention, though this was only to ensure intervention fidelity and accuracy in data recording, and would not be necessary should the intervention run in the absence of scientific study. However, the authors do recommend that one intervention specialist shadow and assist for the first two sessions that the intervention is running. Other daily time commitments included setting up posters and food labels (~ 1 min), chopping fruit for fruit pot servings (~ 10 min), and cleaning food pots after use (~ 5 min). No assessment was made as to the additional time commitment required when compared to the usual lunchtime routine, and this will be addressed in future research.

### Data collection procedure

Data were recorded in April 2017 over two days at baseline and two days at follow-up (three weeks later) in each school. Schools were either visited on a Monday and a Wednesday, or a Tuesday and a Thursday. On these days, researchers (comprised of undergraduate and postgraduate research assistants, and the PhD candidate project lead) arrived at the school around one hour before the lunch period to set up a data collection area in the school cafeteria. One researcher visited participating classrooms to distribute identification stickers and wristbands, and to explain the research to the children. Neutral statements were used to avoid cueing social desirability.

The protocol we used to measure consumption was validated in our previous research [19]. Average food portion weights were calculated based on five servings of every food item available in the cafeteria on each day. At lunchtime, participants were instructed to come to researchers after they had been served their lunch, and again after they finished eating, so that pre- and post-consumption photographs could be recorded for each child.

#### Data processing and coding

##### Consumption estimates from digital photographs

The first author of this paper estimated, using an 11-pont scale (0–100%), how much of each individual food item had been consumed for each child’s lunch. This was then converted into estimated weight consumed (e.g. if 90% of a 56-g portion of baked beans was consumed, then the total weight consumed was calculated as 50.4 g). Total lunchtime fruit, vegetable, fibre, vitamin C, and sugar consumption were then calculated for each participant. An experienced second coder independently calculated consumption for approximately 20% of the data set, to determine inter-rater reliability. Near-perfect levels of inter-rater agreement were achieved (Cohen’s k = .939, CI = .835–.926).

##### Preliminary data analyses

All data were inputted into the IBM Statistical Package for the Social Sciences (SPSS) version 24, including participant number, food item, estimated pre- and post-weight records, estimated weight consumed, first rater percentage estimations, second rater estimations, and agreed estimated weight consumed. Where the first and second coder disagreed on how much of a food item was consumed by 10% or less, the estimation from the first coder was taken, and where they disagreed by more than 10%, the middle value was used.

#### Sample Size Calculations

##### Within groups comparisons

Using an alpha of 0.05, a sample of 176, and two tails, it was identified that a medium effect size (*d* = 0.5) would be detected at a power of 1.

##### Between groups comparisons

Using an alpha of 0.05, a sample of 86 (intervention) and 90 (control), and two tails, it was identified that a medium effect size (*d* = 0.5) would be detected at a power of 1.

##### Effect size calculations

Effect sizes were calculated for each test by dividing the *z* score by the square root of the number of observations, with the subsequent *r* value indicating the magnitude of the effect (.1–.29 = small, .3–.49 = moderate, and ≥ .5 = large effect [28];.

## Results

Daily fruit, vegetable, fibre, vitamin C, and sugar consumption at lunchtime were calculated for each participant, for the two measurement points (T1, baseline and T2, follow-up).

A number of mixed effects repeated measures maximum likelihood regression models with fixed effects (Time) and AR(1) heterogeneous covariance matrix were run to control for any possible clustering effects on the scores for the dependent variables for the intervention condition in SPSS-24 [29]. Preliminary analysis with Generalized Linear Models (GLM) identified no significant effects for the baseline and follow-up scores for the control condition so these findings are unreported.

Model estimates for the mixed effect analyses are presented in Table 1 and Table 2.

**Table 1:** Model estimates for the mixed effects analysis of the intervention conditions’ consumption dependent variables

Dependent Variable	Estimate of Fixed Effect	*Std. Error*	*t*	*p*	ARH1 rho (std. error)	Wald Z (*p*-value)
Fruit	− 9.47	2.28	−4.15	<.001	0.27 (0.10)	2.68 (.007)
Vegetables	1.88	2.62	0.72	.473	0.45 (0.09)	5.25 (.001)
Vitamin C	−2.99	0.88	−3.37	<.001	0.41 (0.90)	4.51 (.001)
Fibre	−0.66	0.13	−4.77	<.001	0.65 (0.06)	10.58 (.001)
Sugar	−2.63	1.40	−1.88	.064	−0.07 (0.11)	−0.65 (.513)

**Table 2:** Model estimates for the mixed effects analysis of the marginal means for the intervention conditions’ consumption dependent variables

Dependent Variable	T1 mean	T2 mean	Mean difference (std. error)	*p*	95% CI
Fruit	7.88	12.35	−9.47 (2.28)	<.001	−14.00 to −4.93
Vegetables	28.72	26.84	1.88 (2.62)	.473	−3.31 to 7.09
Vitamin C	6.60	9.58	−2.99 (0.89)	<.01	−4.74 to −1.23
Fibre	2.55	3.21	−0.66 (0.14)	<.001	−0.94 to − 0.39
Sugar	12.58	15.22	−2.63 (1.40)	.064	−5.42 to 0.16

*Note*: 95% CI = 95% confidence intervals for lower and upper bound from the pairwise comparison for baseline to follow-up

After controlling for any possible group-cluster effects, significant increases were found in the intervention condition for consumption of fruit (*F* (1, 86) = 17.21, *p* = .001), vitamin C (*F* (1, 86) = 11.39, *p* = .001), and fibre (*F* (1, 86) = 22.78, *p* = .001), from baseline to follow-up. No significant changes in consumption were identified in the intervention condition for vegetables (*F* (1, 86) = 0.52, *p* = .473) and sugar (*F* (1, 86) = 3.52, *p* = .064), from baseline to follow-up. Lastly, no sandwich estimate corrections were made because of the small number of clusters in this study, just two schools per condition [30].

**Fig. 1:**
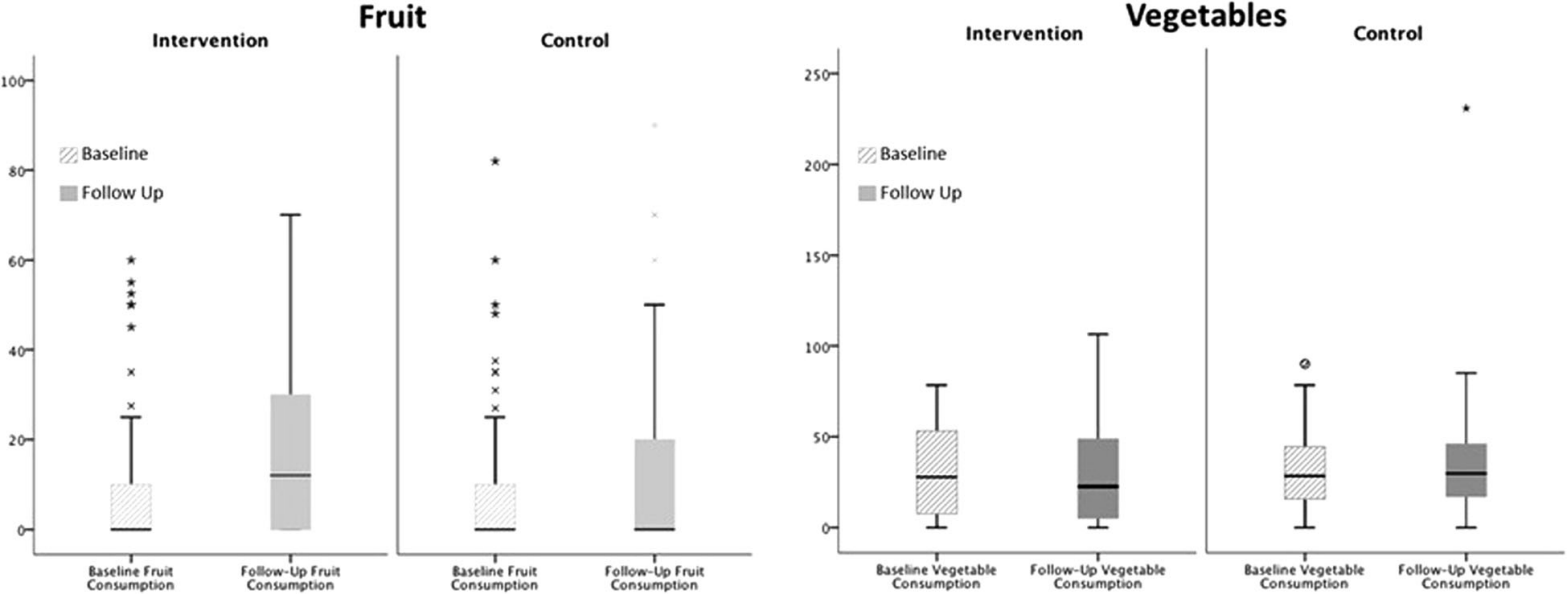
Boxplots showing children’s daily consumption of fruit and vegetables at lunchtime. Medians, interquartile ranges, and distributions of children’s consumption in grams for fruit, fiber and sugar, and milligrams for vitamin C are shown at baseline (striped bars) and follow-up (solid bars) for the intervention and control condition. Key statistics for pairwise within- and between-condition tests are presented to aid interpretation of the data

Figure 1 shows changes in children’s consumption of fruit and vegetables between the two measurement points. The two conditions were matched at baseline (*U* = 3611.5, *p* = .354, *r* = .07). A significant increase in fruit consumption with a moderate effect size (*r* = .43) was observed in the intervention condition, with this effect size being typical of those found in similar literature [31]. By contrast, median consumption did not change in the control condition.

We grouped individual children’s data into three categories: those who ate no fruit; those who consumed less than half of a child-sized portion (20 g); and those who ate more than a half-portion. Table 3 confirms that the increase in fruit consumption due to intervention can be observed across categories.

**Table 3:** Number of children in each fruit consumption category, condition, and measurement point

	Intervention		Control	
Fruit Consumed	Baseline	Follow-up	Baseline	Follow-up
None	63	35	57	58
Less than half a portion (< 20 g)	19	39	29	24
More than half a portion (> 20 g)	6	12	4	8

We examined individual children’s data to establish how many children changed their consumption between baseline and follow-up measurements. Table 4 shows a fairly constant consumption in the control group, where most children ate the same at the two measurement points and small comparable numbers ate either more or less fruit. In the intervention condition the pattern is different, with many more children eating more in the follow-up and fewer eating less. Although not all children benefited from the intervention, many did.

**Table 4:** Number (and percentage) of children in each condition whose fruit consumption increased, remained the same, and decreased, between baseline and follow-up measurements

	Changes in Fruit Consumption		
Children	Increase	No Change	Decrease
Intervention (*N =* 86)	40 (46.5%)	38 (44.2%)	8 (9.3%)
Control (*N* = 90)	17 (18.9%)	57 (63.3%)	16 (17.8%)

Vegetable consumption was matched at both baseline and follow-up, with no significant changes recorded in the intervention schools or control schools over time. Median consumption across measurement points and conditions varied from 22.45 to 29.73 g, with the individual children consuming between 0 and 231 g of vegetables per day.

### Changes in Children’s nutrient intake

The two groups were not matched at baseline, with participants in the control condition consuming significantly more fibre, vitamin C, and sugar than their intervention condition counterparts. This was a result of a higher uptake of nutrient-dense self-serve salad, and additional helpings of leftover food items. Although menus were largely matched at baseline (both pairs of schools were catered by the same company), catering staff in one control school were observed to be comparably more encouraging for children to select self-serve salad, whilst catering staff in the other control school were more likely to encourage second and third helpings of leftovers, than were those in other schools.

**Fig. 2:**
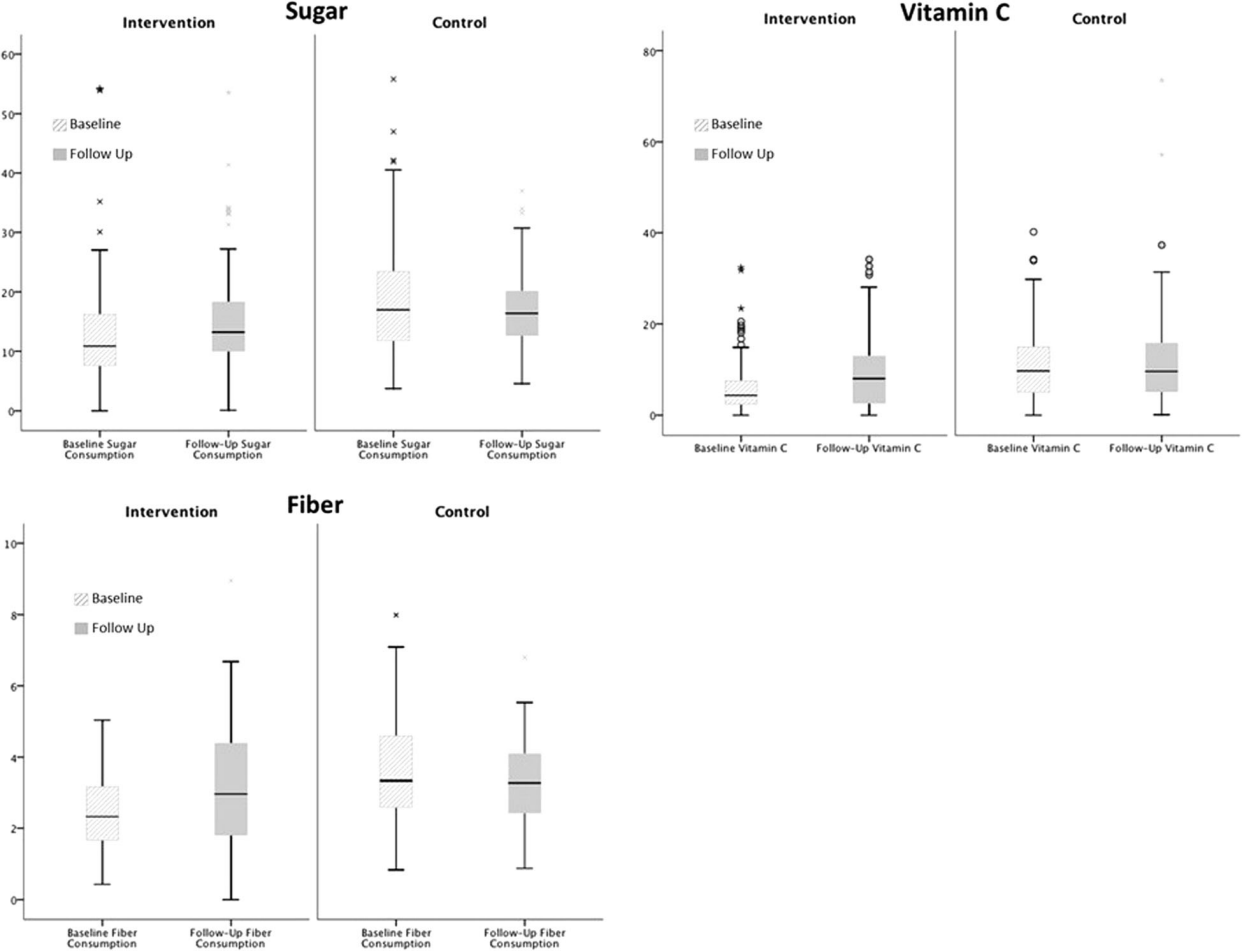
Boxplots showing children’s daily consumption of fiber, vitamin C, and sugar at lunchtime. Medians, interquartile ranges, and distributions of children’s consumption in grams for fruit, fiber and sugar, and milligrams for vitamin C are shown at baseline (striped bars) and follow-up (solid bars) for the intervention and control condition. Key statistics for pairwise within- and between-condition tests are presented to aid interpretation of the data

Figure 2 shows that a significant increase in vitamin C consumption with a moderate effect size was observed between measurement points for the intervention condition, but not the control condition. Levels of vitamin C consumption were matched at follow-up, with consumption levels in the intervention condition rising to the same level as the control condition. A significant increase in fibre consumption with a moderate effect size was observed over time in the intervention condition, whilst a small but significant decrease in fibre consumption was observed in the control condition (though significant due to the highly powered analysis, the median decrease in fibre was .06 of a gram, probably reflecting weekly random variance in consumption). Levels of fibre were matched at follow-up, with consumption levels of the intervention condition rising as the level of consumption in the control condition fell. Finally, though fruit consumption significantly increased, sugar consumption remained stable over time.

A post-hoc calorie intake analysis was conducted in order to identify any influence of increasing fruit consumption on total energy consumption. The two groups were matched at baseline (Intervention *Median* = 340.71, Control *Median* = 339.15, *U* = 3767, *p* = .760, *r* = −.02), however, calorie intake was not matched at follow-up (Intervention *Median* = 378.81, Control *Median* = 378.81, *U* = 3114, *p* = .025, *r* = −.17). The calorie intake of the intervention group remained stable over time (*Z* = −.6, *p* = .548, *r* = −.06), whilst the calorie intake of the control group significantly decreased, with a small effect size, (*Z* = − 2.12, *p* = .034, *r* = −.21). Though a decrease was identified, this represents a difference of only 17 cal, and can be attributed to weekly fluctuations in calorie intake.

## Discussion

This study is the first to present a controlled evaluation of a behavioural nudge intervention designed to increase fruit and vegetable consumption of primary-age children in UK school cafeterias. Within and across condition comparisons based on individual children’s data revealed that their fruit consumption, vitamin C, and dietary fibre intake increased in the intervention schools, but not in the control schools. This finding has significant implications for national-level efforts to improve children’s diets, as recommended by the DOH [5] and for international drives to improve childhood nutrition.

The present study builds upon the existing research in several ways. Many previous investigations situated in the school cafeterias have assessed intervention success using food item selection data, often at the point of sale [12, 15, 32]. This method is convenient and reliable, but without measuring consumption it cannot be ascertained that the intervention was successful. Indeed, some papers reported an increase in target food item selection, but not consumption [32, 33]. The present study was the first to measure children’s consumption using a validated data collection protocol [21], and thus conclusions of effectiveness could be confidently asserted.

It also is the first evaluation of a behavioural nudge intervention in the school cafeteria to measure changes in children’s food consumption at an individual level. We identified that the intervention was successful in increasing fruit consumption from the poorest eaters to those who already consumed adequate levels of fruit. This was manifest in the decreased percentage of children consuming no fruit, whilst the percentage of children eating up to half a portion, and over half a portion, doubled. In addition, we are the first to report nutrient changes over time, with previous nudge studies simply reporting weight or portion changes for target foods. The significant increase in vitamin C consumption recorded in this study has implications for immune system strength [34], whilst increases in fibre promote digestive health [35]. In addition, increases in fruit consumption were not associated with increases in sugar or calorie consumption.

The intervention was effective for fruit but not for vegetable consumption. This is not uncommon in the literature [18, 33], however, there could be multiple reasons for this finding. First, some nudges we employed may have been more effective than others. Participants were visibly enthusiastic about the brightly coloured pots with a selection of different fruits cut into bite-sized chunks, arranged on tiered stands more commonly associated with cake displays. Due to environmental constraints, no such prompts could be used to encourage selection of vegetables and salad. Conversely, re-branding of the fruit and vegetables and colourful advertisements of the “vegetable of the day” may have been much less effective because of the already stimulating nature of the environment. Primary school cafeterias are typically loud and busy, and attractively named vegetables, posters, and “food spikes” may not have been salient. In future studies, we plan to introduce brightly coloured pots with a variety of salad options (e.g., halved cherry tomatoes, cucumber slices, and bell peppers) cut into bite sized chunks, to investigate the effects of this nudge on children’s vegetable consumption. We consider it unlikely that the present vegetable results were due to ceiling effects, because fairly low consumption, about half a child-sized portion on average per day, was recorded in all schools at baseline.

More generally, changes to the choice architecture can only be expected to enable those children who already eat fruit and vegetables to make healthier day-to-day lunchtime choices, but nudges are unlikely to change the behaviour of the remaining children who have not learned to like the target foods. Indeed, looking at the individual children’s data, some children in the intervention schools continued to choose no fruit with their lunch, even though we know that taste preferences for sweet foods favour fruit consumption over vegetable choices that may be bitter in taste [36]. In future interventions, acceptability of a variety of vegetables and fruit could possibly be increased by repeated tasting sessions organised by the school catering team [37].

Another consideration for future development of this intervention is its sustainability. A close engagement with the catering teams is necessary to engender a sense of ownership, which promotes intervention fidelity [38]. Our interactions with the school caterers indicated that the time needed to slice and serve the fruit, and wash the pots afterwards, presented a manageable and acceptable additional workload. However, a large and sustained increase in demand for fruit would present them with an additional cost, which is at present not covered by any national scheme or payment. We are continuing to work in partnership with the school caterers from several boroughs to find solutions to this obstacle to wider implementation, and to develop a simple intervention pack that would enable the other schools to follow suit without the need for involvement of the research team.

## Conclusion

This study presented a low-cost intervention to encourage the consumption of fruit and vegetables in primary-age children. Following changes to the choice architecture of intervention school cafeterias, we observed a significant increase in children’s lunchtime consumption of fruit, vitamin C, and fibre. No changes were observed over time in the control condition, or for children’s consumption of vegetables. These results can be used to inform decision makers, schools, and caterers about simple yet effective behavioural nudge strategies that can improve the poor fruit intake typical of children’s diets.
